# Comparison of Tecar Therapy and Low-Level Laser Therapy Separately and Simultaneously on Clinical Symptoms and Health-Related Quality of Life in Individuals with Type 2 Diabetes: A 3-Month Follow-up Study

**DOI:** 10.5812/ijem-143135

**Published:** 2024-05-05

**Authors:** Mitra Javan Amoli, Khosro Khademi Kalantari, Zeinab Ahmadpour Emshi, Aliyeh Daryabor, Sedigheh Sadat Naimi

**Affiliations:** 1 Student Research Committee, Department of Physiotherapy, School of Rehabilitation, Shahid Beheshti University of Medical Sciences, Tehran, Iran; 2 Department of Physiotherapy, School of Rehabilitation, Shahid Beheshti University of Medical Sciences, Tehran, Iran; 3 Physiotherapy Research Center, School of Rehabilitation, Shahid Beheshti University of Medical Sciences, Tehran, Iran

**Keywords:** Tecar Therapy, Low Power Laser Therapy, Peripheral Neuropathy, Type 2 Diabetes, Neuropathy Symptoms, Quality of Life

## Abstract

**Background:**

Distal peripheral neuropathy (DPN) is a prevalent issue among patients with type 2 diabetes mellitus. Despite the widespread use of low-level laser therapy (LLLT) and limited use of Tecar therapy in physiotherapy for diabetics, the synergistic effect of these two interventions in a long-term follow-up has not yet been determined.

**Objectives:**

This study aimed to compare the effects of Tecar therapy and LLLT separately and simultaneously over a 3-month follow-up period on clinical symptoms and health-related quality of life in individuals with type 2 diabetes and DPN.

**Methods:**

In this double-blind, randomized clinical trial, forty-five individuals with type 2 diabetes (30 women and 15 men) with DPN were randomly assigned to three groups of 15 people: Tecar-on + laser-sham, Tecar-on + laser-on, and laser-on + Tecar-sham. The patients received ten treatment sessions and were followed up for 3-months after the last session. Health-related quality of life was assessed using the WHOQOL-BREF Questionnaire, while clinical symptoms, including pain (measured with a Visual Analog Scale), functional balance (evaluated with the timed-up and go test), and neuropathy symptoms (assessed with the Michigan Questionnaire) were also recorded.

**Results:**

Inter-group comparison after ten sessions revealed that the Tecar-on + laser-sham and Tecar-on + laser-on groups exhibited significant improvement in neuropathy symptoms compared to the laser-on + Tecar-sham group. Even after the 3-month follow-up, these two groups showed lasting improvement in all variables compared to the laser-on + Tecar-sham group (P < 0.05). The Tecar-on + laser-on group demonstrated a more enduring significant effect on pain scores (P = 0.035) compared to the Tecar-on + laser-sham group after the 3-month follow-up. In intra-group comparison, all three groups showed significant improvement in clinical symptoms and health-related quality of life after ten treatment sessions compared to before treatment (P < 0.05). Moreover, after the 3-month follow-up, both the Tecar-on + laser-sham group and the Tecar-on + laser-on group demonstrated a more lasting significant effect in all variables compared to before treatment (P < 0.05). For the laser-on + Tecar-sham group, a more durable improvement in health-related quality of life (P = 0.000) and neuropathy symptoms (P = 0.011) was reported after the 3-month follow-up compared to before treatment.

**Conclusions:**

Although all three groups exhibited significant improvement in clinical symptoms and health-related quality of life in individuals with type 2 diabetes and DPN after ten treatment sessions, the synergistic use of Tecar therapy and LLLT after a long-term follow-up period could lead to more durable therapeutic effects in improving these outcomes for individuals with diabetes.

## 1. Background

Diabetic peripheral neuropathy (DPN) is among the most prevalent types of neuropathy, often affecting multiple peripheral sensory and motor nerves (1). Pain in DPN stems from vessel damage, feeding sensory nerves, and axonal atrophy. This pain significantly impacts patients' mental well-being, sleep, daily activities, and overall health-related quality of life (2, 3). Non-invasive physiotherapy modalities, such as electrical stimulation, low-power laser therapy, infrared waves, and electromagnetic waves, are recommended due to their lack of side effects and prolonged efficacy in pain and neuropathy symptom reduction, often used alongside medication treatments (4).

Low-frequency electromagnetic waves are utilized to alleviate pain and neuropathy symptoms, with reported benefits in musculoskeletal injuries like low back pain (5-7) and achilles tendonitis (8). Tecar therapy, another electromagnetic wave modality, has seen limited studies regarding its effects. Possible physiological mechanisms of Tecar therapy include enhancing cell renewal through tissue oxygenation, accelerating metabolism, releasing endorphins to reduce pain, dilating blood vessels, and improving blood supply function (9). Although Tecar therapy is commonly used for musculoskeletal and sports injuries (10), research on its impact on neuropathy symptoms remains scarce. A prior study noted the effectiveness of combining Tecar therapy with infrared therapy in alleviating pain and sole-foot sensation in individuals with type 2 diabetes. However, the study did not assess the efficacy of Tecar therapy alone (11).

Another study from 2015 suggested that long-wave diathermy with interferential therapy is not recommended as a clinical treatment for patients with chronic neuropathy resulting from chemotherapy (12). Thus, based on the limited research available, there is still no definitive conclusion regarding the efficacy of Tecar therapy in treating diabetic patients, indicating the need for further investigation in this area.

On the other hand, low-level laser therapy (LLLT) has shown broad effectiveness in alleviating various painful conditions and promoting nerve tissue repair. The laser has the potential to biostimulate the nervous system (13, 14), along with other mechanisms such as increasing adenosine triphosphate, releasing endorphins, and exerting anti-inflammatory effects (14, 15). Some researchers have proposed LLLT as a novel treatment approach for diabetic individuals experiencing DPN symptoms, advocating for its inclusion in the peripheral nerve rehabilitation protocol for DPN patients (16-18).

Despite the widespread use of LLLT and the limited utilization of Tecar therapy in diabetic physiotherapy, the synergistic effect of these two interventions has not been established to date. Furthermore, the long-term therapeutic durability of these interventions has not been thoroughly investigated. Given the physiological effects of Tecar therapy, including cell proliferation, tissue repair, and lymph flow improvement (8), as well as the physiological effects of LLLT, such as biostimulation leading to mitochondrial stimulation and increased capillary blood flow (19), conducting a study to examine and compare the duration of effectiveness of each intervention alone and in combination could provide valuable insights.

## 2. Objectives

Due to the chronic nature of diabetes, it is impractical for patients to continuously visit physiotherapy clinics. Therefore, assessing the lasting effects of these treatment methods with follow-up could be effective in the recovery of these patients. The aim of this study was to compare the effects of these two modalities separately and simultaneously with a long-term follow-up of 3-months on clinical symptoms and health-related quality of life in individuals with type 2 diabetes. We hypothesized that the combination of Tecar and laser therapy would have a longer therapeutic effect than either laser or Tecar therapy alone on the clinical symptoms and health-related quality of life of these patients.

## 3. Methods

### 3.1. Participants

This study was a double-blind, randomized controlled trial (IRCT20221105056408N1) with a 3-month follow-up conducted at the physiotherapy clinic of the School of Rehabilitation, Shahid Beheshti University of Medical Sciences, following the approval of the Ethics Committee (ethics code: IR.SBMU.RETECH.REC.1400.122). Forty-five patients (30 women and 15 men) with type 2 diabetes and DPN were randomly assigned to three therapeutic intervention groups: Tecar-on + laser-sham (group 1), Tecar-on + laser-on (group 2), and laser-on + Tecar-sham (group 3). Participant randomization into the three groups was conducted using simple randomization, with individuals referred by an endocrinologist included in the study if they met the eligibility criteria. The inclusion criteria were individuals aged over 50 years with peripheral neuropathy of the lower limbs for at least 6-months, diagnosed with DPN symptoms of the lower limbs by a neurologist via electromyography within three months before the interventions, a body mass index of less than 30, a 3-month average blood sugar (glycosylated hemoglobin) of less than 8.5, and a pain score of 4 or more based on the Visual Analog Scale (VAS) (20). Patients were excluded from the study if they had non-systemic peripheral vascular involvement, diabetic neuropathy symptoms, pregnancy, malignant tumors, coronary artery disease, a pacemaker or mechanical insulin pump, knee arthroplasty, metal plaques in the lower limbs or back, or a history of drug addiction, smoking, or alcohol consumption. Patients who met the inclusion criteria completed and signed a written consent form. Using the PASS program, the required sample size per group was calculated as 15 based on a pilot study involving 5 participants per group, focusing on the pain parameter.

### 3.2. Interventions

#### 3.2.1. Tecar Therapy

**Figure 1.**
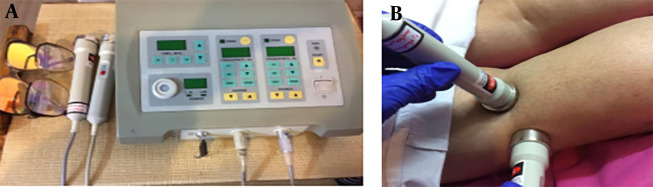
A, the NEW AGE Tecar device used in this study; B, operation with resistance Tecar; C, operation with capacitive Tecar

A Capacitance-resistance Tecar device (TEKRA XCRT, New Age, Italy) was used in this study. It had an output power of 300 watts and capacitive and resistive frequencies of 250 kHz and 500 kHz, respectively, with two metal plates serving as passive electrodes and two capacitive and resistive ergonomic handpieces (Figure 1) (11). The output heat intensity was manually adjustable between 0 - 100%, and for this study, the Tecar device was set at 40 - 50% intensity.

#### 3.2.2. Low-Level Laser Therapy

Low-Level Laser Therapy utilizes light amplification by stimulating radiation emission and has no thermal effect. It is a non-ionizing radiation that does not cause changes in molecular structure or cell damage. The basis of LLLT is the direct effect of biostimulation energy on body cells. In this study, a two-channel laser device (Mustang 2000, manufactured in Russia) with two probes was utilized. The first probe emitted infrared light with a wavelength of 890 nm, power of 15 watts, and frequency of 80 Hz, producing infrared pulse waves. The second probe emitted red light with a wavelength of 630 nm and power of 10 milliwatts, generating continuous waves of red light (21).

### 3.3. Clinical Evaluations

#### 3.3.1. Pain

Pain intensity was assessed using the VAS, an easy, subjective method to evaluate sensory discomfort and its intensity, such as leg pain in DPN. This evaluation tool consisted of a straight 10 cm line with endpoints representing 0 (no pain) to 10 (extreme pain). Participants indicated a number on the line based on the intensity of pain and discomfort felt (22). The VAS Questionnaire demonstrated good reliability, with an ICC of 0.88 reported in a previous study conducted among the Iranian population (23).

#### 3.3.2. Neuropathy Symptoms

Neuropathy symptoms were measured using the Michigan Questionnaire, which includes a 15-item self-administered questionnaire and a physical examination of the lower limbs. The questionnaire assessed foot sensation, including pain, numbness, and temperature sensitivity, with a higher score indicating more neuropathic symptoms. The physical examination included assessments of foot deformities, skin condition, nail or hair abnormalities, calluses, infections, vibration perception, Achilles tendon reflex, and monofilament testing (24). The vibration test was performed by diapason 128 Hz at the designated points with the eyes closed. The monofilament test was evaluated by Semmes Weinstein monofilament of 10 g. Barbosa et al. reported that the Michigan questionnaire was a valid and reliable tool for screening diabetic neuropathy (25). Also, a previous study conducted in Iran stated that the accuracy of the Michigan neuropathy screening instrument is appropriate for individuals with diabetes (26).

#### 3.3.3. Functional Balance

To assess mobility and functional balance, the timed up-and-go test (TUG) was utilized. This test evaluates mobility, balance impairments, and the likelihood of falls in elderly individuals (27). Upon the therapist's command, the patient rose from a chair with a handle, walked 3 meters at normal speed, turned around, returned to the chair, and sat down. The time taken to complete the test activity was recorded using a stopwatch (28). Previous research has indicated that the TUG test is a suitable balance assessment tool for individuals with and without a history of falls, and it is more sensitive in identifying Persian elderly individuals at risk of falling (29).

### 3.4. Health-Related Quality of Life

The WHOQOL-BREF self-reported questionnaire was employed to assess health-related quality of life. This 24-question survey measures four domains: Physical health (7 items), psychological health (6 items), social relationships (3 items), and environmental health (8 items). Scores ranging from 4 to 20 are obtained for each domain separately, with 4 representing the worst condition and 20 indicating the best condition within the domain. These scores are then linearly transformed to a 0 - 100 scale (30). A total score encompassing all domains was calculated for the final analysis. The validity and reliability of the Persian version of the WHOQOL have been confirmed in previous studies involving Iranian elderly individuals (31).

### 3.5. Evaluation Method

Before commencing treatment, the evaluator measured fasting and 3-month average blood sugar levels, as well as demographic characteristics. The patient completed the Michigan Questionnaire, focusing on relevant parts of the clinical tests, including evaluation of achilles tendon reflex and vibration sensation (using a 128 Hz tuning fork) and assessment of sole-foot sensation using a 10 g monofilament. Subsequently, pain intensity was assessed using the VAS. Following this, the patient completed the WHOQOL-BREF Questionnaire. Finally, the functional balance test was performed, and the time taken was recorded. Rest periods were allowed between tests. After the initial evaluation of clinical tests and health-related quality of life, patients were randomly assigned to one of the groups.

To ensure therapist blinding, the main researcher did not assign patients to the three groups; this task was carried out by a research assistant who was a physiotherapy expert. Additionally, the therapist remained unaware of the evaluation results at three different times: Before and after ten intervention sessions and after 3-months of follow-up. For patient blinding, individuals were kept unaware of whether they received real or sham stimulation. Clinical variables and health-related quality of life were assessed by the main researcher, who was a physiotherapist. Furthermore, the statistician was completely unaware of the grouping of the individuals.

To initiate treatment in the Tecar-on + laser-on group (group 2), patients comfortably lay prone with a pillow under their abdomen and ankles. Initially, LLLT was administered using two probes with different wavelengths at four points in the L2 - L4 lumbar region and two points in the popliteal region on both sides, with each point treated for 2 minutes (totaling 16 minutes). To optimize time and prevent patient fatigue, two laser wavelengths were simultaneously applied by the two probes. Following this intervention, the patient rested for 10 minutes before commencing Tecar therapy.

For Tecar therapy, the inactive (metal) electrode, coated with cream, was positioned on the abdominal area. Subsequently, the active electrode with a 6 cm diameter was slowly moved using the cream for 10 minutes on the lumbosacral area. The same procedure was repeated using the resistance method for 5 minutes, maintaining the same intensity in the lumbosacral region. Treatment then proceeded bilaterally with the capacitance method in the popliteal area, with the inactive electrode coated in cream positioned in the upper area of the patella. The capacitive active electrode was then slowly moved in the popliteal region for 10 minutes, followed by the application of the resistance method for 5 minutes using a specialized resistance probe.

For the Tecar-on + laser-sham (group 1) and laser-on + Tecar-sham (group 3) groups, the same procedure was repeated with the device turned on while the Tecar or laser was actually turned off (sham).

All participants underwent evaluation in the morning, with the room temperature maintained between 25 and 27 °C. In all three groups, a total of 10 treatment sessions were conducted over three sessions per week. Assessments related to pain, neuropathy symptoms, and functional balance were performed three times: Before the start of treatment, after the completion of 10 sessions, and during a 3-month follow-up after the 10th session. Evaluation of health-related quality of life occurred twice: Before treatment and during the 3-month follow-up. Additionally, after a 15-minute rest during treatment sessions, exercise therapy, including strengthening, stretching, and weight-bearing exercises, was carried out for 15 minutes in all three groups. Patients were reminded to perform exercises at home and were monitored via phone during the follow-up period.

### 3.6. Statistical Analysis

Statistical analysis involved the use of the Kolmogorov-Smirnov test to assess the normality of data distribution in the groups. Differences among the three groups regarding demographic and clinical characteristics at baseline were compared using one-way ANOVA or chi-square, depending on the variable type. Inter-group comparison of normally distributed variables of clinical symptoms and health-related quality of life over time was conducted using two-way mixed repeated measures ANOVA, and Bonferroni's method was applied for intra-group pairwise comparison. All analyses were performed using SPSS 20 software, with a significance level set at 0.05.

## 4. Results

**Figure 2.**
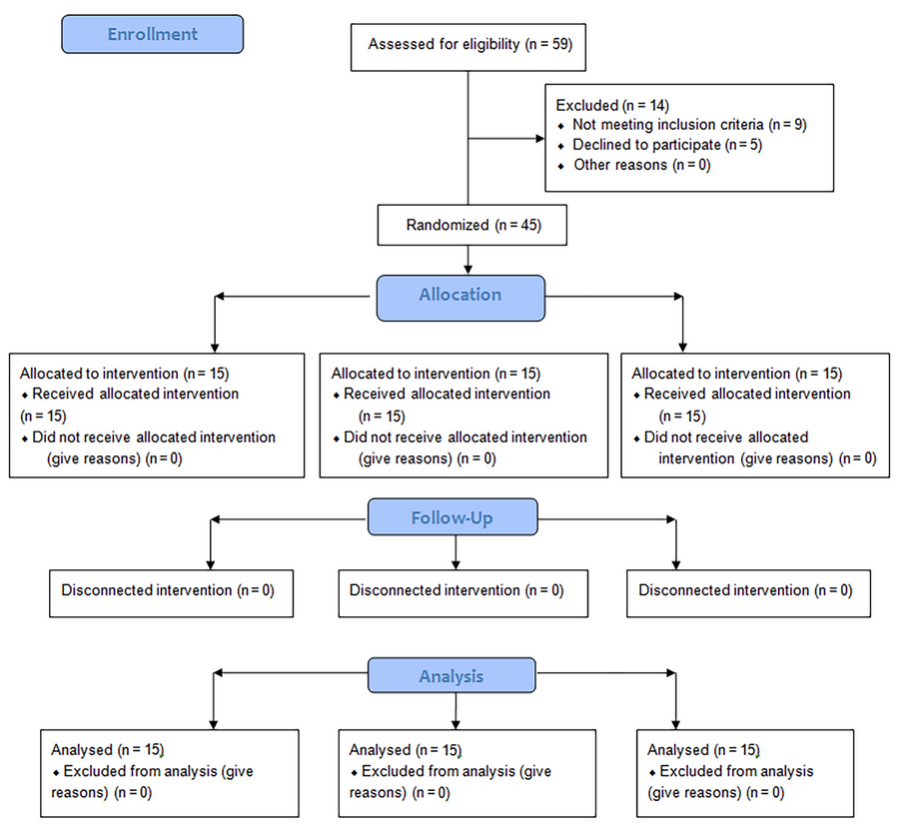
CONSORT flowchart

Table 1 displays the demographic and clinical characteristics of the participants, showing no significant differences among the three groups in these variables (P > 0.05). Fifteen individuals in each group completed the study and were included in the analysis (Figure 2). Table 2 presents the mean and standard deviation of clinical variables and health-related quality of life, along with inter-group comparisons after 10 intervention sessions and after 3-months. Intra-group comparison results are provided in Table 3. 

**Table 1.** Demographic Characteristics of Patients

Variables	Tecar-on + Laser Sham (n = 15)	Tecar-on + Laser-on (n = 15)	Laser-on + Tecar Sham (n = 15)
**Gender (female/male)**	11/4	9/6	10/5
**Age, (y)**	63.93 (7.44)	64.46 (7.69)	68.60 (7.31)
**Body Mass Index, (kg/m** ^**2**^ **)**	26.54 (1.58)	26.15 (1.12)	25.29 (2.34)
**Duration of diabetes,( y)**	13.47 (1.5)	13.13 (1.92)	13 (1.30)
**Duration of neuropathy, (y)**	2.66 (1.23)	2.86 (1.06)	3.20 (0.86)
**HbA1c, (%)**	7.06 (0.6)	7.13 (0.67)	7.32 (0.71)
**fasting blood sugar, (mg/dL)**	132 (8.81)	132.32 (9.35)	129.73 (8.64)

**Table 2.** Mean Outcome Measures and Comparison Between Groups After 10 Intervention Sessions and After 3-Months ^a^

Variables	Group 1 ^b^ (n = 15)	Group 2 ^c^ (n = 15)	Group 3 ^d^ (n = 15)	Comparison Between Groups After 10 Sessions (P-Value)			Comparison Between Groups After 3-Months (P-Value)		
				Groups 1 & 2	Groups 1 & 3	Groups 2 & 3	Groups 1 & 2	Groups 1 & 3	Groups 2 & 3
**Pain **				0.917	0.823	0.585	0.035 ^e^	0.000 ^f^	0.000 ^f^
Before intervention	6.47 ± 1.06	6.60 ± 1.12	6.27 ± 1.22						
After 10 sessions	1. 67 ± 1.11	1.80 ± 0.77	1.47 ± 0.83						
After 3-months	1.73 ± 0.79	0.80 ± 0.77	6.13 ± 1.30						
**Functional balance **				0.770	0.025 ^e^	0.116	1.000	0.000 ^f^	0.000 ^f^
Before intervention	14.26 ± 1.55	16.25 ± 4.37	18.84 ± 9.42						
After 10 sessions	10.33 ± 1.20	11.25 ± 2.25	13.94 ± 5.73						
After 3-months	9.99 ± 1.23	9.96 ± 1.58	17.68 ± 8.53						
**Health-related quality of life**				-	-	-	0.991	0.014 ^e^	0.010 ^e^
Before intervention	184.72 ± 34.79	180.50 ± 23.75	185.22 ± 33.16						
After 10 sessions	-	-	-						
After 3-months	226.95 ± 33.83	228.37 ± 24.27	193.93 ± 32.57						
**Neuropathy symptoms **				0.271	0.000	0.000 ^f^	0.891	0.000 ^f^	0.000 ^f^
Before intervention	10.76 ± 0.72	10.30 ± 1.08	10.43 ± 0.59						
After 10 sessions	4.86 ± 0.99	4.33 ± 1.02	6.93 ± 0.75						
After 3-months	3.63 ± 1.14	3.40 ± 2.02	10.33 ± 0.64						

^a^ Values are expressed as mean ± SD.

^b^ Group 1: Tecar-on + laser sham.

^c^ Group 2, Tecar on + laser-on.

^d^ group 3: Laser-on + Tecar sham.

^e^ P < 0.05.

^f^ P < 0.001.

**Table 3.** Intra-group Comparison Using Repeated Measure Test

Groups			P-Value			
			Pain	Functional Balance	Health-Related Quality of Life	Neuropathy Symptoms
**Tecar-on + laser sham**	Before intervention	After 10 sessions	0.000 ^a^	0.000 ^a^	-	0.000 ^a^
	Before intervention	After 3-months	0.000 ^a^	0.000 ^a^	0.000 ^a^	0.000 ^a^
	After 10 sessions	After 3-months	1.000	0.000	-	0.013 ^a^
**Tecar on + laser on**	Before intervention	After 10 sessions	0.000 ^a^	0.000 ^a^	-	0.000 ^a^
	Before intervention	After 3-months	0.000 ^a^	0.000 ^a^	0.000 ^a^	0.000 ^a^
	After 10 sessions	After 3-months	0.000 ^a^	0.000 ^a^	-	0.256
**Laser-on + Tecar sham**	Before intervention	After 10 sessions	0.000 ^a^	0.001	-	0.000 ^a^
	Before intervention	After 3-months	0.493	0.011^b^	0.000	0.247
	After 10 sessions	After 3-months	0.000 ^a^	0.001 ^b^	-	0.000 ^a^

^a^ P < 0.001.

^b^ P < 0.05.

### 4.1. Inter-Group Comparison

**Figure 3.**
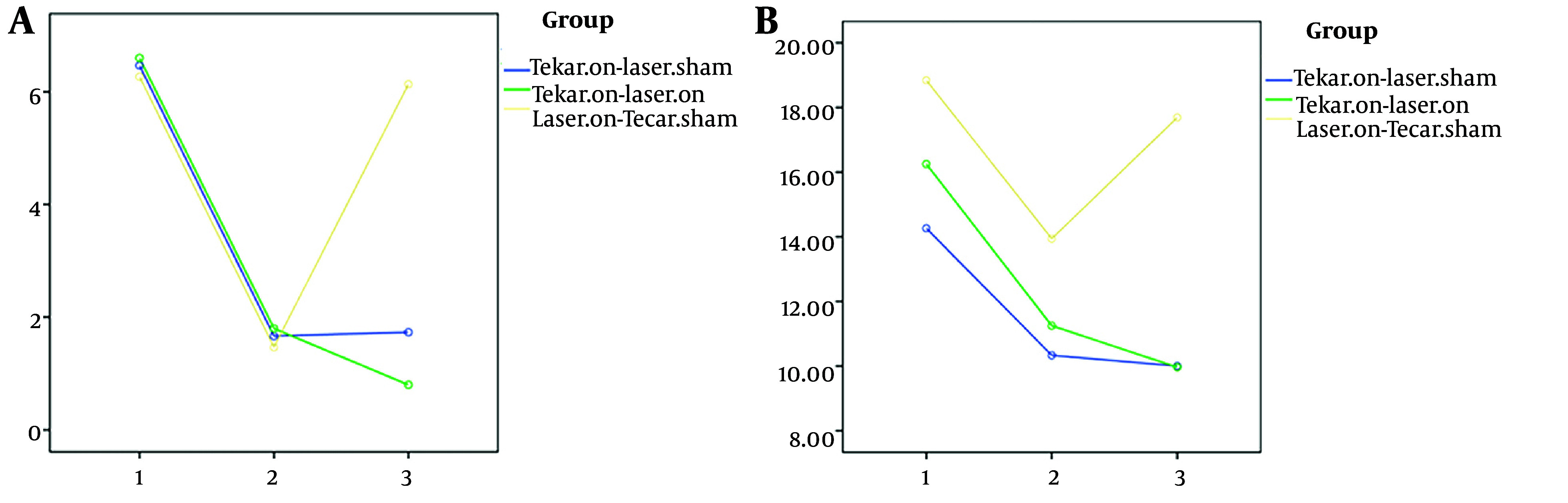
A, pain; B, functional balance; 1, before the intervention; 2, after 10 intervention sessions; 3, after 3-months

**Figure 4.**
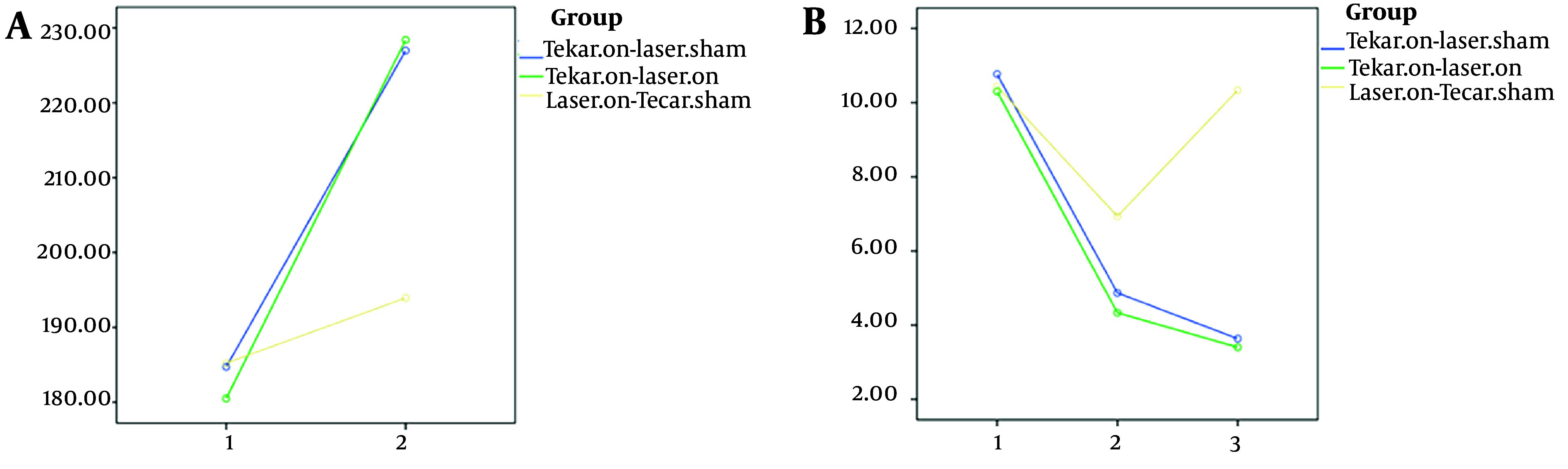
A, quality of life; and B, neuropathy symptoms; 1, before the intervention; 2, after 10 intervention sessions; 3, after 3-months

As shown in Table 2, significant differences were observed among the three groups in two variables: Functional balance (Figure 3) and neuropathy symptoms (Figure 4) after 10 intervention sessions, while no significant difference was found in pain scores. Both the Tecar-on + laser-sham and Tecar-on + laser-on groups exhibited improved neuropathy symptoms after 10 intervention sessions compared to the laser-on + Tecar-sham group. Additionally, the Tecar-on + laser-sham group showed a significant improvement in functional balance compared to the laser-on + Tecar-sham group after 10 sessions.

Even after a 3-month follow-up, a significant therapeutic effect was observed for all four variables (Figures 3 and 4) in terms of reducing functional balance time, pain scores, and neuropathy symptoms, as well as increasing health-related quality of life scores in the Tecar-on + laser-on and Tecar-on + laser-sham groups compared to the laser-on + Tecar-sham group, indicating their sustained effects (Table 2 P < 0.05). Moreover, a significant lasting effect was observed in pain reduction for the Tecar-on + laser-on group compared to the laser-on + Tecar-sham group after 3-months of follow-up (P = 0.035).

### 4.2. Intra-group Comparison

In all three groups, there was a significant improvement in functional balance after ten intervention sessions and also after 3-months of follow-up compared to before the intervention (Table 3 P < 0.001). For pain and neuropathy symptoms, significant improvements were observed in the Tecar-on + laser-on and Tecar-on + laser-sham groups after ten sessions and also after 3-months of follow-up compared to before the intervention (Table 3 P < 0.001). Although there was a significant improvement in pain and neuropathy symptoms after ten sessions compared to before the intervention for the laser-on + Tecar-sham group, the therapeutic effect of these variables did not reach statistical significance after 3-months of follow-up. Regarding health-related quality of life, there was a significant lasting effect on the scores of the WHOQOL-BREF Questionnaire after the 3-month follow-up compared to before the intervention for all three groups (Figure 4). 

## 5. Discussion

Damage to peripheral nerves manifests as burning sensations and numbness in the lower limbs, particularly in the legs. These neuropathic symptoms often worsen at night, causing sleep disturbances in individuals with diabetes, and if left untreated, may progress to complete numbness of the legs (32). The findings of this study regarding neuropathy symptoms indicated that both the Tecar-on + laser-on and Tecar-on + laser-sham groups exhibited a significant reduction in these symptoms after ten sessions compared to before the intervention. Furthermore, the sustained improvement in neuropathy symptoms persisted after 3-months in these two groups compared to the laser-on + Tecar-sham group. Bosi et al. (33) investigated the effects of frequency-modulated electromagnetic neural stimulation with variable frequencies ranging from 1 - 50 Hz on the foot vibration perception threshold in diabetic neuropathy over 10 treatment sessions and a 4-month follow-up. Their results demonstrated statistically significant changes, including a decrease in the number of insensitive points to the Semmes–Weinstein monofilament and a decrease in foot vibration perception threshold after the 4-month follow-up. Similarly, Niajalili et al. (11) studied the effect of capacitive therapy on diabetic neuropathy and reported a significant decrease in the Michigan Questionnaire score after 10 sessions and a 6-week follow-up, which aligns with the findings of the present study. These studies proposed that the significant improvement in neurological symptoms of the lower limbs following Tecar therapy may be attributed to increased blood circulation and vascular endothelial growth in the treated area. Therefore, the improvement in neuropathy symptoms observed with capacitive and resistive Tecar therapy in this study may be explained by the mechanisms of cellular regeneration and accelerated release of oxygen from tissue hemoglobin (11).

Furthermore, the results regarding neuropathy symptoms showed that laser-on + Tecar-sham resulted in a significant decrease in the Michigan Questionnaire score after 10 intervention sessions. In a non-follow-up study, Kumar CG et al. (24) investigated the effect of LLLT on painful DPN and reported a decrease in Michigan score and an increase in vibration perception threshold after 10 sessions. The observed improvements in the present study are consistent with the findings of Kumar CG et al. after ten sessions of LLLT intervention. It is likely that LLLT stimulates the release of cytokines and growth factors responsible for capillary dilation and the formation of new capillaries in blood flow, thereby reducing neurological disorders (24).

Regarding pain, the findings showed a significant reduction in pain scores in two groups, Tecar-on+ laser-sham, and Tecar-on+ laser-on, after ten intervention sessions and after 3-months of follow-up. It can be concluded that the use of Tecar both alone and in combination with laser resulted in a long-lasting, therapeutically significant effect on this outcome in patients with type 2 diabetes. These findings align with the studies of Bosi et al. (33) with a 4-month follow-up and Niajalili et al. (11) with a 6-week follow-up. They stated that the reduction in pain could be attributed to the activation of the analgesic mechanism resulting from the thermal and non-thermal effects of Tecar therapy. The thermal effects of Tecar are based on vasodilation, which improves blood flow and oxygenation in damaged tissues. The non-thermal effects of Tecar create an electromagnetic current in the target tissue, causing ions in the tissue to move faster, thereby increasing the activity of the neurovascular system associated with positive effects such as reducing swelling and inflammation, ultimately reducing pain. Therefore, low-intensity Tecar can result in the transmission of radio frequency waves and cell proliferation in damaged tissues.

On the other hand, the results of the inter-group comparison demonstrated a significant improvement in pain reduction in the combined Tecar and laser group compared to Tecar-only or laser-only at the follow-up time. Therefore, the simultaneous use of both interventions could create a better lasting effect in pain relief. It can be inferred that capacitive therapy, along with resistance therapy, due to its strong effects and impact on high resistance tissues, as well as laser therapy, due to its anti-inflammatory effects resulting in improved blood flow and increased peripheral microcirculation, were able to maintain a long-lasting therapeutic effect on pain reduction compared to the use of Tecar-only or laser-only.

According to the findings, the laser-on+ Tecar-sham group showed a significant improvement in pain and neuropathy symptoms only after ten intervention sessions. However, its therapeutic effect was not sustained after the 3-month follow-up. Although previous studies (16, 19, 34) demonstrated that laser intervention alone was able to improve pain or neuropathy symptoms in individuals with type 2 diabetes after several intervention sessions, none of them conducted a long-term follow-up after the end of laser application, and the results were limited to the immediate evaluation of these parameters after the completion of the laser application sessions, which aligns with the present study. The potential physiological effects of LLLT, such as creating a biostimulation effect on the nervous system, lead to the stimulation of intracellular mitochondria, resulting in the production of adenosine triphosphate (ATP) and growth factors. This could increase capillary blood flow and oxygenation of damaged nerve tissues, thereby relieving pain and clinical symptoms of peripheral neuropathy (24). Therefore, these effects were significant only after ten intervention sessions.

On the other hand, it can be assumed that since LLLT does not cause thermal effects and has no impact on increasing the flexibility of vascular tissues and their surrounding tissues, it has not been able to sustain therapeutic effects in the long term. This sustainability may be achieved with more sessions of LLLT aimed at reducing pain and improving neuropathy symptoms.

Elderly individuals with DPN often encounter difficulties in walking speed, body stability during gait, and balance issues due to sensory impairments and muscle weakness, which can negatively impact their functional balance and health-related quality of life (35). The results of the current study regarding functional balance and health-related quality of life demonstrated significant improvements after ten intervention sessions, with the continuation of their therapeutic effects observed during the 3-month follow-up in all three groups for these variables. In inter-group comparison, the Tecar-on + laser-on and Tecar-on + laser-sham groups exhibited a sustained therapeutic effect for these variables after 3 months of follow-up compared to the laser-on + Tecar-sham group.

Regarding the effect of laser therapy after ten sessions, the findings of this study are consistent with previous research. Chatterjee et al., investigating the effect of 12 sessions of deep tissue laser therapy on painful neuropathy in individuals with type 2 diabetes, reported improvements in function, walking speed, and health-related quality of life (27). Similarly, Sahier et al. reported an enhancement in health-related quality of life in individuals with DPN after 12 sessions of LLLT use (36). It's worth noting that neither of these previous studies included a follow-up period after the sessions. In contrast, in the present study, we conducted a 3-month follow-up. The sustained therapeutic effect observed after three months with the use of Tecar both alone and in combination with a laser may be attributed to the increased flexibility of soft tissues and the continuation of exercises during the follow-up period, resulting in improved functional balance and health-related quality of life. Additionally, none of the previous studies incorporated exercise therapy. Therefore, it is conceivable that engaging in exercises alongside Tecar and laser interventions can contribute to longer-term recovery after the completion of intervention sessions.

One limitation of the current study is that it only included individuals over 50 years of age, which restricts the generalizability of the results to other age groups. Additionally, there was limited control over participants' diabetes management throughout the study, including factors such as nutrition, timing of diabetes medications, and other environmental influences. Other limitations include the absence of a control group with similar interventions and the lack of post-intervention electromyography. Future research could explore similar interventions in individuals with type 1 diabetes and compare the effects of capacitive Tecar with resistance Tecar-on outcomes in individuals with diabetes. Furthermore, conducting studies with a greater number of sessions and longer follow-up periods would provide valuable insights.

### 5.1. Conclusions

In conclusion, while all three groups demonstrated significant improvements in clinical symptoms and health-related quality of life among type 2 diabetic individuals with DPN after ten intervention sessions, the synergistic use of Tecar therapy and LLLT showed enhanced durability of therapeutic effects over the long term.

## Data Availability

The dataset presented in the study is available on request from the corresponding author during submission or after its publication.

## Clinical Trial Registration Code:

Clinical Trial Code: IRCT20221105056408N1.
